# Impact of a Comprehensive Anti-Smoking Program at a Regional University Hospital and Predictive Variables of Being a Smoker among Hospital Workers

**DOI:** 10.3390/ijerph17228432

**Published:** 2020-11-14

**Authors:** Antonio Ranchal-Sánchez, Esperanza Romero-Rodríguez, Jose Manuel Jurado-Castro, África Ruiz-Gandara, Manuel Vaquero-Abellán

**Affiliations:** 1 Department of Nursing, Pharmacology and Physiotherapy, Faculty of Medicine and Nursing, University of Cordoba, 14071 Córdoba, Spain; en1vaabm@uco.es; 2 Maimonides Biomedical Research Institute of Cordoba (IMIBIC), Reina Sofia University Hospital, University of Cordoba, 14004 Córdoba, Spain; espe_mrr@hotmail.com (E.R.-R.); juradox@gmail.com (J.M.J.-C.); 3 Department of Community Health Sciences, Boston University School of Public Health, Boston, MA 02118, USA; 4 Department of Applied Economics, University of Seville, 41018 Seville, Spain; africaruiz@us.es

**Keywords:** tobacco use prevention, smoking cessation, evaluation studies, youth and young adults, adult smokers

## Abstract

The objective of this study was to evaluate the impact of a comprehensive anti-smoking health program conducted over twelve years at a regional university hospital in southern Spain. Prevalence of tobacco was compared retrospectively using data collected during occupational health assessments (*n* = 4291). Bivariate and logistic regression analyses were carried out to evaluate tobacco consumption differences according to age, sex, professional category, and workplace building. The results show a reduction in the active smoking rate among hospital staff evaluated (from 22.8% to 19.8%) with significant differences between non-health and health workers. Accumulated smoking consumption fell to 13.45 ± 14.60 packs/year with men presenting a higher consumption (*p* < 0.001). The predictive variables of tobacco use were sex (greater consumption among men, *p* = 0.021), number of cigarettes (greater consumption among professionals who smoked less than 1 pack/day, *p* < 0.001), and time smoking (greater use among professionals with more than 10 years smoking, *p* < 0.001). There was a higher rate of staff smokers at the hospital building with a majority of mental health inpatients. This study provides a practical example of making the optimum use of digital medical records in the evaluation of a comprehensive anti-smoking health program.

## 1. Introduction

In Spain, smoking causes an average of 51,870 deaths annually [1], so action is required to combat this addiction. In this context, the anti-smoking laws [2] have favoured the development of health programs to encourage smoking cessation among healthcare workers. A sizeable percentage of professionals (e.g., doctors, nurses, physiotherapists, technicians) are health workers and they should set a good example to the patients they attend in their daily work. It would therefore be desirable for most hospitals to implement these programs.

Tobacco use among health professionals is a matter of concern to the scientific community [3,4]. A recent meta-analysis [5] concluded that the prevalence of smoking among health workers remains high, which is why urgent action is required, as they constitute the front line when dealing with smoking in their patients [5]. Anti-smoking intervention programs are therefore necessary in health centres, especially hospitals [6]. A prior study [7] analyzed the results of a program carried out among 24,000 workers in 21 Chinese hospitals from 2009 to 2010. A reduction from 14.8% to 10.7% was achieved in the prevalence of smoking among the staff, which illustrates one important knock-on benefit of turning a hospital into a smoke-free zone. Xiao et al. [7] found a greater proportion of male smokers but did not show sex differences in smoking behaviours or by occupation. Martínez et al. [8] in a 45 Catalan hospital meta-analysis in physicians, nurses, assistant nurses, administrative and other staff did not also show sex differences in smoking behaviours and by occupation.

Running an anti-smoking action program requires the involvement of a multidisciplinary team, led by management, in order to tackle the problem comprehensively through different lines of work [8], ranging from monitoring compliance with the regulations to imposing suitable fines to penalise non-compliance. In addition, there should be an on-going campaign of notices and constant information-giving and awareness-raising measures targeting hospital visitors. Continuous training should also be provided to teach professionals the basic steps in quitting smoking [9], a smoking cessation clinic should be made available, and anti-smoking treatment should be offered to hospitalised patients, which should be followed up during primary care. Furthermore, it is important for health centres which design, conduct, and maintain health programs to be made more accountable to society [10]. The Spanish General Public Health Law 33/2011 establishes in Article 3.e that “the performance and results of public health actions must be evaluated” [11]. Here, smoking cessation programs can be carried out and their impact over time can be evaluated by comparing the changing smoking prevalence rates among the hospital staff. To achieve this, resources such as digital medical records, which every hospital has, can be useful. In fact, the analysis of digital medical records to support clinical research using massive data analysis plays a key role in the Spanish Strategy for Science and Technology and Innovation 2013–2020, as part of the “Health, Demographic Change and Well-being” [12] challenge. 

In this context, the first aim of this work was to evaluate the impact, in terms of prevalence, of an anti-smoking health program conducted over twelve years at a regional university hospital in southern Spain. The second aim was to examine, through digital medical records, the association between smoking (and measures of smoking duration) and independent variables, such as sex, professional category, and workplace (building area) in a university hospital and to assess the influence of predictive variables on smoking in hospital workers.

## 2. Materials and Methods 

### 2.1. Design

A cross-sectional observational descriptive study was carried out to evaluate the impact of an anti-smoking health program based on the data collected in the routine health check-up with the staff of a regional university hospital in southern Spain.

### 2.2. Population and Sample

The working population of the hospital was 5882 workers at the established cut-off point in January 2020, and after the data were filtered to exclude duplicate cases, those not meeting the inclusion criteria, and those whose variables of tobacco consumption were incomplete, a sample size of 4291 was fixed to analyse smoking habits.

The inclusion criteria were being a health or non-health worker with a contract in the regional hospital and being below 70 years of age (the maximum legal working age for one of the professional categories-medical area specialist) with a contract at the regional hospital. Associated University medical and nursing staff were also included. 

All the subjects signed the obligatory form to give their informed consent to be included in the study. The study was carried out following the Declaration of Helsinki, and the protocol was approved by Ethics Committee (Record 239, Ref. 2788), using the ARS2 code to comply with the Data Protection Law. The STROBE statement guidelines for observational studies were followed [13].

### 2.3. Period and Data Collection Method

The programme, initiated in 2008, was developed through different lines of action. On the one hand, a punishment system enforced compliance, with imposed fines targeted at both staff and other smokers who broke the rules. On the other hand, signs and notices being displayed using specially designed diagrams and placed wherever needed according to maps. In addition, proactive steps were taken to increase awareness and to provide information and training. Mostly nursing staff were trained in basic intervention methods for tackling smoking and help was offered by the occupational health unit at specially organised clinics to smokers among the hospital staff who voluntarily decided to quit smoking. Nicotine replacement therapy (NRT) was prescribed for hospital patients who met the requirements established by the company, following the current protocols. Varenicline and bupropion were also prescribed to smoking hospital workers in the occupational health unit.

The current smoking prevalence rate was collected in January 2020 from the database supplied by the Andalusian Health Service (SAS) central facilities. The current smoking prevalence rate was compared with that obtained in the same hospital working population in 2013 [14] and the 2016 prevalence rate taken from the population of Medical Internal Resident (MIR) Specialists [15]. The data on tobacco consumption were obtained from Winmedtra, a corporate computer program used in the SAS when carrying out the obligatory occupational health assessments [14].

### 2.4. Variables Analysed

The variables analysed for tobacco consumption were: smoking (smoker, non-smoker, ex-smoker); year of starting smoking and year of quitting (for ex-smokers); average daily cigarette consumption using the pack-year index for smokers and ex-smokers. Workers who consumed at least one cigarette a day were considered smokers. Workers who had not smoked for at least six months were considered ex-smokers [15]. In addition, the database included information about sex, age, professional category and workplace (i.e., the different buildings which made up the hospital complex). The professional categories with a smaller number of workers were grouped together with those with an equivalent academic degree and/or who carried out the same essential tasks (specialist technicians, managers, administrative staff, canteen staff, laundry workers, maintenance staff) to avoid an excessive number of categories. In order to make contrasts, staff were also classified into health workers and non-health workers (e.g., caretakers).

Other data were also collected on the steps taken as part of the comprehensive program, including money raised by imposing fines, number of trained personnel, amount of NRTs provided to hospitalised patients, informative campaigns, public notices, and awareness-raising activities.

### 2.5. Statistical Analysis

The statistical analysis was performed using SPSS V.17.0 and EPIDAT V.3.1 software. Central and dispersion measures were calculated for the quantitative variables and frequencies for the qualitative ones. Age was converted into an ordinal qualitative variable in two categories: 50 years old or younger, which is close to the mean age at the hospital [16], and over 51 years old.

To compare tobacco consumption, the 2013 [14], 2016 [15], and 2020 prevalence rates at the same hospital were used; the difference in proportions was calculated with a 95% confidence interval (CI). Pearson’s test was used to compare the consumption trend and to analyse the results of the 2020 study (*p* ≤ 0.05). The data are shown as the difference of means (MD) ± their standard deviation (SD), or by comparison of different percentages.

A bivariate analysis was carried out to verify the existence of differences in tobacco consumption according to age, sex, professional category, and workplace building using the Student’s t (parametric test used to compare differences between sample means). Next, a multivariate analysis was performed using a logistic regression considering the dependent variable of tobacco consumption. Age, sex, professional category, and workplace building were included in the regression model as independent variables. The model’s goodness of fit was evaluated using the Hosmer–Lemeshow test. 

Finally, a binary regression logistic model was performed to verify which variables were independently associated with the smoking habit. The variables included in the regression model were age, sex, type of professional, workplace, average daily cigarette consumption, and time smoking. Those variables whose *p* value with the Wald test was >0.05 were eliminated, thereby obtaining the most parsimonious model. Cox, Snell, and Nagelkelke determination coefficients were calculated.

## 3. Results

### 3.1. Impact, in Terms of Prevalence, of the Anti-Smoking Health Program

During the 2020 selection, 19.8% of the staff were active smokers (95% CI: 18.60–21.01%); 21.6% (95% CI: 20.22–22.70%) were ex-smokers; 58.6% (95% CI: 57.10–60.07%) had never smoked, which means that 80.2% did not smoke at the time of the study (95% CI: 78.98–81.39%). In the 2013 study, 22.8% (95% CI: 20.79–24.81%) of the staff were active smokers and 77.2% (95% CI: 75.19–79.21%) were non-smokers and ex-smokers. 

Comparing sex and accumulated consumption, there were also significant differences (*p* < 0.001) when comparing the cumulative consumption data from 2013 with 2020, with 16.5 ± 14.10 pack-years in 2013 vs. 13.45 ± 14.60 pack-years in 2020 (comparison of two means of independent samples).

### 3.2. Association between Smoking (and Measures of Smoking Duration) and Independent Variables

During the 2020 cut-off point the majority of the total working population at the hospital were women, with the majority being health staff, as can be seen in Table 1. The mean age of the staff evaluated was 49.9 years (σ 10.6) (95% CI 49.7–50.2), with a range from 22 to 69 years.

#### 3.2.1. Association between Smoking and Sex, Age, Occupation and Workplace Variables.

In the 2020 bivariate analysis between smoking and sex, a higher percentage of smokers was male than female, although these differences were not significant (Table 2). When the smoking habits of health staff vs. non-health staff were compared, significant differences were obtained (*p* < 0.001), which shows that there were more smokers among the non-health staff (Table 2). In terms of smoking according to the workplace building of the hospital complex, the data from “Peripheral hospital” (*p* = 0.004) are conspicuous, with the highest proportion of staff who smoke (41.1%). It should be noted that most of the inpatients admitted to this “Peripheral hospital” hospital are mental health patients, as the hospital houses a therapeutic community (Table 2). 

Table 3 shows significant differences (*p* < 0.001) between MIRs, Nursing Internal Residents (NIRs) and the other staff in the 2020 analysis. The prevalence of active smokers among MIRs was 5.6% (compared to 20.9% for the rest of the staff). 

As shown in Table 4, with the exception of “unassigned staff”, whose exact professional category is unknown, the lowest rate of smokers was among the medical MIR staff, followed by the management staff linked to the University, and the Medical Area Specialists (MASs), all with a rate below 12%. It is noticeable that the MIR staff was the most representative sample of the total working population analysed, since 99% completed the survey. All in all, the medical staff had the lowest percentages of smokers compared with the different professional categories at the hospital.

Significant differences (*p* < 0.001) were also found about age, showing a higher average age for smokers (53.11 ± 8.37) and ex-smokers (53.26 ± 8.63) compared with non-smokers (47.52 ± 11.45). 

#### 3.2.2. Association between Measures of Smoking Duration and Sex

Lastly, quantifying the number of cigarettes and the pack-year index in smokers and ex-smokers, we found a 13.45 ± 14.60 packs/year of accumulated smoking consumption in smokers as shown in Table 5. We observed significant differences in accumulated smoking (*p* < 0.001): 13.15 ± 8.03 in men, compared to 11.07 ± 86.87 pack/years in women.

#### 3.2.3. Influence of Predictive Variables on Smoking in Hospital Workers

The independent variables related to the smoking habit, by means of logistic regression analysis, were: sex (greater among men, *p* = 0.021), professional category (greater among assistants and administrative staff vs. nurses, *p* = 0.101), time smoking (greater among professionals with less than 10 years smoking vs. 0 years smoking (*p* = 0.003), and those with more than 10 years smoking vs. 0 years smoking, *p* < 0.001), and the number of cigarettes (greater among professionals who smoked less than 1 pack/day vs. those who did not smoked, *p* < 0.001). Between 65.8% and 88.6% of the variation of the dependent variable is explained by the variables included in the model (Table 6).

### 3.3. Other Results

Additionally, other results for the data at different stages of the comprehensive anti-smoking program were EUR 12,030 collected by imposing fines and 1921 workers trained in basic smoking cope.

## 4. Discussion

The present study reveals the impact of a multidisciplinary program of anti-smoking measures conducted over twelve years, showing a reduction in the rate of active smoking of the hospital staff evaluated at a regional university hospital. This was based, above all, on the increase in non-smoking healthcare staff compared to non-health staff, and particularly in young people, where the majority of newly qualified doctors (medical and nursing residents) were non-smokers. Our findings also show an increase in ex-smokers and a decrease in accumulated tobacco consumption. Furthermore, the logistic regression analysis shows people who are assistants, administrators, or men and who smoke less than one tobacco pack/day and who have been smoking for more than ten years are more likely to be smokers in the analysed hospital.

The anti-smoking program, initiated by the occupational medicine unit, tackled the problem of smoking in the workplace in a comprehensive, multidisciplinary approach, with different lines of action. On the one hand, a punishment system enforced compliance, with fines imposed which added up to over EUR 12,000, targeted at both staff and other smokers who broke the rules, despite numerous signs and notices being displayed using specially designed diagrams and placed wherever needed. In addition, proactive steps were taken to increase awareness, and to provide information and training. During this period, around 2000 workers, mostly nursing staff, were trained in basic intervention methods for tackling smoking, and help was offered at specially organised clinics to smokers among the hospital staff who voluntarily decided to quit smoking. 

Many health promotion and prevention programs have been initiated, including those that address tobacco addiction, but few were sustained over long periods of time and even fewer were evaluated. However, this should be mandatory in Spain, not only because the General Public Health Law 33/20113 explicitly states this, and also all over the world for reasons of efficiency, to justify to the public the human and material resources used. One way to assess the impact of anti-smoking action plans is by measuring the rates of staff who smoke (which logically should decrease) over a period of time and comparing this with other data from national and regional surveys. In addition to quantifying, with objective data, the results of the action plan, another specific aim of the work was to compare the prevalence rates of active smokers among the staff over a period of six years. The results obtained indicate a reduction of three points in the prevalence rate of smokers among the staff at the hospital in question, which fell from 22.8% in 2013 [14] to 19.8% in January 2020. Furthermore, the percentage of ex-smokers increased by 1.8 points, from 19.8% [14] to 21.6%. These data may have been influenced by the clinic run by the occupational health unit since 2008 to encourage smoking cessation among staff.

As regards treatment, Malone et al. [17] mention an increase in the supply of NRT to patients by nurses after their hospital intervention. We think it is a wise move to target the nursing staff, not only because, as can be seen in Table 4 of our study, it is the largest group in a hospital, but also because of they represent a high percentage of smokers within the healthcare staff (Table 4). This is a crucial issue, considering that nursing staff should set the best example, starting with the supervisors, especially in those units which care for the most vulnerable patients, such as those with a severe mental illness (SMI). It is noteworthy that, of all the hospital areas analysed in our study (Table 2), it is the workplace with the most mental health inpatients proportionally (“the therapeutic community of peripheral Hospital”), where the highest rates of smoking are found among the staff. To the best of our knowledge, this is the first time that indicators have been used to compare smoking in the different areas which form a hospital complex. This idea could be useful to guide policies and available resources in the future. As for mental health, it is known that there is a higher prevalence of smoking consumption and a more intense smoking habit among patients suffering from an SMI [18]. Therefore, further research is needed to explore the factors associated with tobacco use in hospital staff who work at mental health centres.

There are examples in the literature about networks of hospitals in smoking cessation [19]. As regards tackling smoking in hospitals across Spain, the region of Catalonia has been a pioneer in creating smoke-free hospital networks. Martínez et al. (2020) addressed, in a multicentre study, the patient’s perception of tobacco control [20]. They concluded that most of the respondents looked up to health professionals as models for smoking cessation, so support should be provided to empower them [20]. Michael Fine, in his publication “the Public Health Value of Hospitals” [21], suggests that hospital investments, which prove ineffective, should be employed to fund public health interventions, among which he cites smoking cessation [21], thus offering added value to the society whose funds it depends on. Another study, also conducted in the United States, indicates that policies aimed at increasing the use of tax revenues for tobacco cessation measures among staff can offset the tobacco-related burden among individuals with problems of substance abuse [22].

Another specific aim of this work was to assess age and smoking habits. One fact of note is the significant difference obtained when comparing the average age of non-smokers and smokers among the staff, particularly among increasingly aging populations of workers such as those at large hospitals [16], which is in line with the demographic evolution of the Spanish population. In this context, the fact that over 80% of hospital staff do not smoke is largely due to the fact that the majority of the young people who join the workplace have never smoked. This is a particularly positive sign, given the well-known financial savings of prevention, as opposed to diagnosis, treatment, and prognosis. This occurs especially in young healthcare staff such as NIRs/MIRs. In fact, the results obtained in the MIRs (only 5.6% of daily smokers) are even an improvement on the low prevalence obtained in the 2016 study carried out at the same hospital [15] when a prevalence of 6.5% was obtained for MIR staff [15]. This percentage is well below the 17.6% figure for young people who smoke in the general Spanish population [23], and significantly less than 27.4% of daily smokers for the age group of 18 to 24 years shown in the fifth Andalusian Health Survey [24]. According to these data, it seems that there is an increased awareness among young health workers which conditions their habits of consumption. 

Moreover, the reduction in tobacco use could also be due to the information and training campaigns carried out with the students of the Faculty of Medicine and Nursing (which is attached to the hospital), as part of the program. A Spanish study published in 2004 [25], before the implementation of anti-tobacco legislation and carried out among 3840 sixth-year medical students, indicated “a lower prevalence of smokers among these students compared to other populations” [25], despite the figure of 27% for students who smoked [25]. The authors attribute this fact to better habits and higher motivation. This study also concludes that one worrying figure was “the large number of students who start smoking in medical schools” [25]. The low prevalence obtained in our study could be a reflection of the impact that the legislation against this addictive substance is having on the new generations of physicians who are being trained under its protection, as well as on the associated programs. This is no trivial matter, due to its knock-on effect among this group, the vanguard of newly qualified doctors, and the mirror in which their older colleagues are reflected, those “eternal contemplators” who show greater reluctance to quit the smoking habit. In contrast, Bourbon et al. [26], in a study carried out among young French medical students, observed a higher prevalence of smoking (18.9%) than that of our study and also associated severe nicotine dependence with the consumption of anti-anxiety medication among them [26]. These discrepancies point to the need for studies to evaluate the impact of anti-tobacco laws on medical and/or nursing students from different countries all over the world.

Another factor in the relationship between graduated health staff and smoking is shown in the results of our study, which show significant differences between health staff and non-health staff. It can be seen that the lower the level of education among the staff, the higher the smoking rate. In particular, the highest percentages of smokers are found in the categories of caretakers, assistant nurses, and catering staff. Given that both caretakers and assistant nurses are workers who come into contact permanently with the patients, anti-smoking measures should target them, so that, once awareness is raised, they can in turn promote smoking cessation [27]. 

As far as sex is concerned, we have found no significant differences when comparing the global figure for this variable with the subgroup of smokers, where, as in most health centres, women are in a clear majority (75% of staff at the hospital we evaluated are women). However, there are significant differences when comparing the average accumulated tobacco consumption by sex as men consume a greater amount of tobacco packs/year. There were also significant differences (*p* < 0.001) when comparing the data on cumulative tobacco consumption from 2013 to 2020, with reduced consumption in both sexes. Therefore, in 2020, the average accumulated tobacco consumption among smokers decreased to an average of 13.45 ± 14.60 packs/year. This figure is positive since long-term smokers are more reluctant to quit smoking. Nilan et al. [5] concluded in their 2020 meta-analysis that, as regards sex, in countries with high economic resources, male health workers tend to have a lower prevalence of smoking compared to those in the general population, while the rate is similar in women. However, among those health workers from countries with medium-low and low resources, they tend to have a similar or even higher prevalence than those of the general population [5]. This shows how much influence the economic and social environment has, even in smoking, among health professionals.

Regarding the limitations of this work, the percentage of missing data must be mentioned. Regarding the registration of the smoking habits in the clinical history, Xiao et al. [7] mention in their work the difficulty of implementing a routine to register the smoking status of patient. Another limitation is the quantification of the smoking habit into sporadic, occasional or “weekend” smokers and how to label them, since the computer application only records the variables of smokers, ex-smokers, and non-smokers, and those considered non-smokers may actually be sporadic smokers. This is relevant given the relationship that occasional smoking seems to have with mortality [28]. In any case, the same smoking criteria were maintained in all three studies, in 2013, 2018 and 2020. Another limitation of the study is the lack of pre-intervention data prior to before 2008 (when the program was implemented); therefore, we should be cautious when we attribute any change in smoking outcomes to the anti-smoking program implemented in the hospital, since the pre-intervention data were not available. 

On the other hand, we consider that one of the strengths of this work is to show the results of an interdisciplinary program which was conducted for over a decade, with data obtained from digital medical records, and highlighting resources and tools used on a daily basis. The systematic recording of relevant data on the digital medical record could also serve as support when monitoring other variables needed to keep the current pandemic under continuous surveillance. 

Some practical suggestions are derived from our experience in this work. About the unregistered smoking habit data, the nursing staff of the units, instead of doctors, should carry out the recording of its systematically during the first round of occupational health surveillance to avoid “missing data”. On the other hand, it would be necessary for experts to clarify the variables “sporadic”, “occasional”, and “weekend smokers” and how to label them, since the computer application usually only records the variables of smokers, ex-smokers, and non-smokers, and those considered non-smokers may actually be sporadic smokers. This is just a small indication of the advisability of including the label of “occasional smoker” when registering the status in medical records, so that this more exact standardisation will permit us in future to compare the indicators better than with the categories used in our study, as well as to introduce in the electronic clinical history the last nicotine-containing products consumed such as e-cigarettes, JUUL, and other heated tobacco products. Additionally, the model from the logistic regression points out the smoker staff members to act on with priority in the analysed hospital.

Furthermore, the results with data obtained from digital medical records highlight the cost effectiveness from resources and tools used on a daily basis. The systematic recording of relevant data on the digital medical record could also serve as support when monitoring other variables needed to keep the current pandemic under continuous surveillance. In addition, they could serve as a basis for auditing both practitioners at the individual level, as well as units/services or hospitals at a collective level, and the anti-smoking programs for promoting health in the workplace. In this context, our work could serve as a guide, giving a practical example of the retrospective evaluation of anti-smoking measures.

## 5. Conclusions

On balance, the results of the multidisciplinary comprehensive program of anti-smoking measures conducted over twelve years, which included one consultation for smoking cessation, show a reduction in the rate of active smoking of the hospital staff evaluated. This was based, above all, on the increase in non-smoking healthcare staff compared to non-health staff, and particularly young people—the majority of medical internal residents were non-smokers, with age being a relevant variable. The percentage of ex-smokers also increased and the accumulated tobacco consumption by smokers decreased. As for those who continued to smoke, it should be noted that the hospital branch with the highest rate of smoking was the one with a majority of patients who had a severe mental illness. Some innovations of this work include a comparison of smoking in the different areas that make up the hospital, and a regression logistic model that predicts the smoking habit in hospital staff. We also propose some practical suggestions regarding the regular recording of smoking status by nursing staff, the inclusion of the variable of “occasional smoker” in the medical history, which must be clarified by experts to unify this status with “occasional” and/or “weekend” smoking, in addition to the inclusion of the last nicotine-containing products in the digital medical records. Additionally, this work provides an example of how to evaluate health promotion programs using everyday tools, along with the idea that individual and collective actions should be audited, based on the data recorded in the digital clinical records. This is even more true with online monitoring, which is set to become a major tool for implementing public health measures, including those which tackle the problem of smoking addiction.

## Figures and Tables

**Table 1:** Distribution of the population according to sex, professional category and workplace (*n* = 5882).

Demographic and Occupational Variables	Frequency (*n*)	Percentage (%)
Age (years) (mean (σ))	49.9 (10.6)	
**Sex**		
Female	4392	74.7
Male	1490	25.3
**Health staff vs. non-health staff**		
Non health staff	1467	24.9
Health staff	4415	75.1
**Workplace**		
General Hospital	3414	58.0
Provincial Hospital	1184	20.1
Maternity-Children’s Hospital	815	13.9
Peripheral Hospital	80	1.4
Local Specialist Centre	87	1.5
Government Building	214	3.6
Local Dialysis Centre	1	0.0
Regional Blood Transfusion Centre	59	1.0
Not assigned	28	0.5

**Table 2:** Smoking habit according to sex, occupation, and workplace in the 2020 sample analysed.

	Smoking Habit			Total (*n* = 4291)	*p* Value
	Non-Smoker *n* (%)	Smoker *n* (%)	Ex-Smoker *n* (%)		
**Sex**					
Female	1919 (58.9)	643 (19.7)	697 (21.4)	3259	0.764
Male	595 (57.7)	207 (20.1)	230 (22.3)	1032	
**Occupation**					
Non-Health	538 (52.6)	237 (23.2)	247 (24.2)	1022	<0.001
Health	1976 (60.4)	613 (18.8)	680 (20.8)	3269	
**Workplace**					
General Hospital	1430 (58.0)	499 (20.2)	538 (21.8)	2467	0.004
Provincial Hospital	500 (58.3)	176 (20.5)	181 (21.1)	857	
Maternity Hospital	390 (61.1)	107 (16.8)	141 (22.1)	638	
Peripherical Hospital	24 (42.9)	234 (41.1)	9 (16.1)	56	
Local Specialist	36 (58.1)	12 (19.4)	14 (22.6)	62	
Government Centre	94 (62.7)	25 (16.7)	31 (20.7)	150	
Transfusion Centre	19 (51.4)	82 (1.6)	10 (27.0)	37	
Not assigned	21 (87.5)	0 (0.0)	3 (12.5)	24	

**Table 3:** Comparison between Medical Internal Residents (MIRs), Nursing Internal Residents (NIRs) and the other staff (*n* = 4291).

Professional Category	Smoking Habit			Total (*n* = 4291)	*p* Value
	Non-Smoker *n* (%)	Smoker *n* (%)	Ex-Smoker *n* (%)		
Nursing NIR	11 (68.8)	2 (12.5)	3 (18.7)	16	<0.001
Medical MIR	273 (90.2)	17 (5.6)	11 (3.6)	301	
Rest of staff	2230 (56.1)	831 (20.9)	913 (23.0)	3974	
Total	2514	850	927	4291	

MIR, Medical Internal Resident; NIR, Nursing Internal Resident.

**Table 4:** Smoking habit according to professional category of the hospital staff evaluated.

Professional Category	Smoking Habit			Total
	Non-Smoker *n* (%)	Smoker *n* (%)	Ex-Smoker *n* (%)	
Nurse	647 (58.4)	207 (18.7)	254 (22.9)	1108
Specialist nurse	37 (64.9)	6 (10.5)	14 (24.6)	57
Nurse Supervisor	18 (46.2)	7 (17.9)	14 (35.9)	39
Nursing Internal Resident (NIR)	11 (68.8)	2 (12.5)	3 (18.8)	16
Assistant nurse	381 (46.5)	252 (30.8)	186 (22.7)	819
Physiotherapist	14 (63.6)	6 (27.3)	2 (9.1)	22
Specialist technician	157 (57.3)	55 (20.1)	62 (22.6)	274
Specialist Pharmacist	20 (58.8)	6 (17.6)	8 (23.5)	34
Caretaker	136 (45.0)	92 (30.5)	74 (24.5)	302
Medical Internal Resident (MIR)	273 (90.7)	17 (5.6)	11 (3.7)	301
Medical Area Specialist (MAS)	345 (71.4)	40 (8.3)	98 (20.3)	483
Non-MAS Doctor	34 (54.8)	9 (14.5)	19 (14.5)	62
Management	54 (61.4)	10 (11.4)	24 (30.6)	88
Staff linked to University	10 (76.9)	1 (7.7)	2 (15.4)	13
Administrative Staff	183 (56.0)	63 (19.3)	81 (24.8)	327
Canteen Staff	61 (49.6)	36 (29.3)	26 (21.1)	123
Laundry Staff	44 (59.5)	13 (17.6)	17 (23.0)	74
Maintenance Staff	37 (49.3)	20 (26.7)	18 (24.0)	75
Other graduate staff	8 (47.1)	5 (29.4)	4 (23.5)	17
Receptionist & other tasks	11 (61.1)	21 (1.1	5 (27.8)	18
Executive Staff	12 (80.0)	1 (6.7)	2 (13.3)	15
Unassigned Staff	21 (87.5)	0 (0.0)	3 (12.5)	24
Total	3144	850	927	4291

**Table 5:** Measurement of smoking habit among smokers and ex-smokers.

Smoking Habit	Years Smoked (Mean ± SD)	*p* Value	Cigarettes/Day (Mean ± SD)	*p* Value	Smoking Index (Mean ± SD)	*p* Value
Smoker (*n* = 850)	21.10 ± 16.49		11.58 ± 7.23		13.45 ± 14.64	
Men (*n* = 207)	21.08 ± 16.28	0.957	13.15 ± 8.03	0.001	15.93 ± 16.70	0.008
Women (*n* = 643)	21.15 ± 16.55		11.07 ± 6.87		12.67 ± 13.83	
Ex-smoker (*n* = 927)	17.52 ± 12.09		11.05 ± 9.76		11.96 ± 13.65	
Men (*n* = 230)	17.55 ± 12.24	0.695	13.90 ± 11.21	0.001	15.01 ± 16.00	0.001
Women (*n* = 697)	17.51 ± 12.04		10.11 ± 9.05		10.95 ± 12.63	

SD: Standard Deviation.

**Table 6:** Variables associated with smoking. Logistic regression final model.

Variables	B	*p*	OR	CI 95%
**Sex**				
Men vs. women	0.45	0.021	1.57	1.07–2.29
**Professional category**				
Physicians vs. nurses	−0.80	0.003	0.45	0.26–0.76
Assistants vs. nurses	0.47	0.040	1.60	1.02–2.52
Administrative staff vs. nurses	0.44	0.121	1.56	0.89–2.74
Other vs. nurses	−0.09	0.667	0.91	0.58–1.42
**Time smoking**				
Less than 10 years vs. 0 years	2.31	0.003	10.09	2.20–46.15
10 years or more vs. 0 years	5.62	<0.001	274.94	64.51–1171.68
**Number of cigarettes**				
Smoking < 1 pack/day vs. 0 cigarettes	6.65	<0.001	776.01	313.69–1919.69
Smoking > 1 pack/day, but < 2 pack/day vs. 0 cigarettes	40.86	1	0.00	0.00–0.00
Smoking > 2 packs/day vs. 0 cigarettes	35.35	1	0.00	0.00–0.00

Dependent variable: smoking habit; Cox and Snell determination coefficient = 0.658 and Nagelkerke determination coefficient = 0. 886. B = Beta Coefficient; OR = Odds Ratio.
